# Robust Extrinsic Calibration of Multiple RGB-D Cameras with Body Tracking and Feature Matching

**DOI:** 10.3390/s21031013

**Published:** 2021-02-02

**Authors:** Sang-ha Lee, Jisang Yoo, Minsik Park, Jinwoong Kim, Soonchul Kwon

**Affiliations:** 1 Department of Electrical Engineering, Kwangwoon University, 20 Kwangwoon-ro, Nowon-gu, Seoul 01897, Korea; kcv456@kw.ac.kr (S.-h.L.); jsyoo@kw.ac.kr (J.Y.); 2 Electronics and Telecommunications Research Institute (ETRI), Daejeon 34129, Korea; pms@etri.re.kr (M.P.); jwkim@etri.re.kr (J.K.); 3 Department of Smart Convergence, Kwangwoon University, 20 Kwangwoon-ro, Nowon-gu, Seoul 01897, Korea

**Keywords:** RGB-D sensor, Azure Kinect, feature matching, computer vision, image processing, calibration, signal processing

## Abstract

RGB-D cameras have been commercialized, and many applications using them have been proposed. In this paper, we propose a robust registration method of multiple RGB-D cameras. We use a human body tracking system provided by Azure Kinect SDK to estimate a coarse global registration between cameras. As this coarse global registration has some error, we refine it using feature matching. However, the matched feature pairs include mismatches, hindering good performance. Therefore, we propose a registration refinement procedure that removes these mismatches and uses the global registration. In an experiment, the ratio of inliers among the matched features is greater than 95% for all tested feature matchers. Thus, we experimentally confirm that mismatches can be eliminated via the proposed method even in difficult situations and that a more precise global registration of RGB-D cameras can be obtained.

## 1. Introduction

Recently, RGB-D cameras such as Azure Kinect DK [1,2,3] and RealSense D400 series [4,5], have been commercialized. In addition, applications using RGB-D cameras including object pose estimation, 3D reconstruction, and camera localization have been actively investigated. Among RGB-D cameras, Azure Kinect DK [1,2,3] shows excellent performance suitable for Kinect series. Azure Kinect DK [1,2,3] operates based on time of flight, which is a method to calculate the depth between a camera and an object by measuring the round-trip time of a light signal provided by a laser, such as LIDAR. It is possible to obtain good depth information though Azure Kinect DK [1,2,3] and use it in various applications.

However, the global registration between RGB-D cameras is still a challenging task. The global registration is the type of pose estimation using cameras. As the distance or the rotation between cameras is increased, the pose estimation becomes more difficult. There are many reasons behind this problem. The main reason is mismatches within matched pairs. In order to apply the global registration, feature points are generally matched between cameras. However, there are many mismatches within matched pairs. In order to remove these mismatches, post-processing, such as random sample consensus (RANSAC) [6,7], is required. This type of post-processing shows lower performance when the number of mismatches is high. In this paper, we propose a global registration method using body tracking and refinement registration with geometrical information. The rest of the paper is organized as follows. We describe the related works and the proposed method in Section 2 and Section 3, respectively, and experimental results and conclusions are presented in Section 4 and Section 5, respectively.

## 2. Related Works

There are a large number of methods, which implement point cloud registration. The previous methods usually use a global registration and registration refinement, which is called a local registration. Go-ICP [8] is based on a branch-and-bound (BnB) scheme that searches the entire 3D motion space SE(3). By exploiting the special structure of *SE*(3) geometry, novel upper and lower bounds are derived for the registration error function. Fast global registration [9] operates on candidate matches that cover the surfaces. The objective is optimized to align the surfaces and disable false matches. It is more than an order of magnitude faster than prior global registration algorithms and is much more robust to noise. In this paper, we use human body tracking for global registration. Human body tracking, also known as human pose estimation, is a method of detecting and tracking human skeletons. It is applied to game and human action recognition, and so on. OpenPose [10] uses part affinity fields (PAFs) to learn to associate body parts with individuals in the image. The bottom-up system achieves high accuracy and real-time performance, regardless of the number of people in an image. DensePose [11] establishes dense correspondences between a surface-based representation of the human body and an RGB image. There has been a method to detect 3D poses of closely interative humans from multi-view RGB images [12]. The method provided by Azure Kinect DK [13] detects 3D human skeleton from a depth image. It provides the 3d position and orientation of each joint. Thus, it is possible to transform a camera coordinate system into each joint coordinate system. Similarly, Garau et al. [14] proposed an unsupervised automatic framework for calibration outside the camera. It uses an optimized 3D human mesh recovery from a single image.

There are several techniques of camera extrinsic calibration in computer vision. Su et al. [15] proposed a method to calibrate the RGB-D camera networks by using a spherical calibration object. It shows that the approach outperforms other techniques based on planar calibration objects. Kwon et al. [16] use a circular region detection for a spherical calibration. It is used to reduce errors caused by incorrect sphere centers. Iterative k-closest point algorithms [17] consist of two algorithms for calibrating RGB-D cameras. The first algorithm refines the pose parameters to minimize the cost function. The second algorithm regards the measured depth values as variables and minimizes the cost function to obtain refined depth values. Methods based on RGB images usually operate based on feature points. In general, feature detection algorithms detect feature points in RGB images [18]. Feature points are usually detected at the prominent parts such as corners, edges, and so on. The feature points detected though the feature detection procedures are described in logically different ways based on the unique patterns represented by the neighboring pixels. There are various methods of feature detection. The feature detector of the scale-invariant feature transform (SIFT) [19] is based on the difference of Gaussians (DoG) operator, which is an approximation of the Laplacian of Gaussians (LoG) filter. Feature points are detected by searching local maxima with DoG at various scales of images. Additionally, it uses Taylor series expansion of scale-space to obtain more precise locations of extrema, and if the intensity at an extremum is lower than a threshold value, it is rejected. Therein, a 2 × 2 Hessian matrix is used to compute the principal curvature. A STAR feature detector is derived from a center surround extrema (CenSurE) feature detector [20]. It uses an approximation of the LoG filter. The circular shape of the mask is replaced by an approximation that preserves rotation invariance and uses integral images for efficient computation. It operates scale-space by applying masks of different size. Recently, as deep learning has been actively studied, feature detection methods based on deep learning have been proposed [21,22,23]. They have better performance than computer-vision-based methods. TILDE [22] proposed a learning-based approach to detect repeatable keypoints under drastically changing weather and lighting conditions. Superpoint [23] proposed a homographic adaptation which is a multi-homography approach for boosting interest point detection repeatability and performing cross-domain adaptation.

After the feature detection, the feature points should be matched between two cameras to analyze the relation. This is called feature matching. Feature matching is processed by calculating a feature descriptor on a feature point and by comparing similarities of each feature point between two cameras. The feature descriptor encodes the feature point into a series of numbers and acts as a sort of numerical fingerprint that can be used to differentiate one feature from another. Ideally, the feature descriptor should be invariant under scale, rotation, brightness, and so on. There are many methods of feature descriptor calculation. The descriptor of the SIFT [19] extracts a 16 × 16 neighborhood around the feature point, representing a total of 128 binary values. The SIFT [19] is robustly invariant to rotation, scale, and so on, but it requires high computational cost. The speed up robust features (SURF) method [24] is inspired by the SIFT [19]. Generally, its descriptor is represented by a total of 64 binary values. However, it can be expanded to a total of 64 binary values. The main goal of the SURF [24] is to overcome the main weakness of the SIFT [19] though the use of the Haar wavelet approximation. The binary robust invariant scalable (BRISK) method [25] detects feature points with the adaptive and generic corner detection based on the accelerated segment test (AGAST) algorithm and the FAST [26,27]. Additionally, the descriptor of the BRISK [25] identifies the characteristic direction of the feature point for the rotation invariance. It is represented by a binary string and uses the hamming distance to match the feature points. The oriented FAST and rotated BRIEF (ORB) method [28] detects feature points with the FAST [26,27] similar to the BRISK [25]. As the descriptor of the binary robust independent elementary features (BRIEF) [29] is highly unstable against rotation, a modified BRIEF descriptor has been employed. It is a very fast recognizer with good viewpoint invariance. Thus, it has been widely applied to simultaneous localization and mapping (SLAM) [30,31,32]. There are also descriptors based on deep learning that show good performance [21,23,33]. There are a plenty of methods that apply a registration refinement to RGB-D images. The point-to-plane ICP [34] uses a new variant based on uniform sampling of the space of normals. Colored point cloud alignment [35] is a method to optimize a joint photometric and geometric objective that locks the alignment along both the normal direction and the tangent plane. They extend a photometric objective for aligning RGB-D images to point clouds, by locally parameterizing the point cloud with a virtual camera. As 3D features have more mismatches than 2D feature points, they are usually mixed with 2D feature points, e.g., in the SIFT [19] or ORB [28]. However, no matter how well feature matching works, mismatches will be present. A mismatch causes bad registration. Many methods of mismatch removal have been proposed. Among them, the most widely used method is based on the RANSAC [6,7]. Despite its effectiveness in terms of low noise and low number of mismatches, the RANSAC exhibits slow convergence and low accuracy, making it more difficult to sample a good set generated during feature matching. Other approaches are based on the M-estimation, which replaces the least square problem with robust costs that are less sensitive to mismatches [36,37,38]. Methods of robust rotation search based on the RANSAC or M-estimation do not guarantee good performance. In this paper, we propose a method of mismatch removal in a set generated during feature matching in order to improve the registration. This method uses the global registration information to unify a pair of features matched between two cameras into the same 3D coordinate system. Thereafter, mismatches are eliminated when the L2 distance between a pair of the matched features is larger than the threshold.

## 3. Proposed Methods

**Figure 1:**
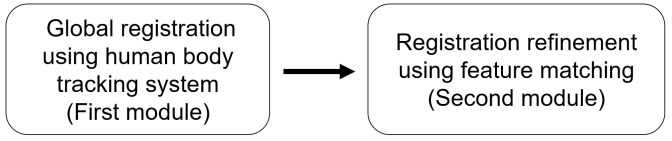
Flowchart of the proposed method.

In this paper, we propose a global registration method based on feature matching between RGB-D images. We use Azure Kinect DK released by Microsoft. The proposed method consists of two modules. A flowchart of the method is shown in Figure 1.

In the first module, the global registration is performed by using a human body tracking system; we use the body tracking API provided by Azure Kinect SDK. The body tracking API is based on a deep learning network. It provides us with a rough estimation of the position between two RGB-D cameras. In this module, we evaluate the global registration information between two cameras with some errors.

In the second module, a registration refinement procedure is performed by using feature matching to reduce the error of the global registration information. This is described in detail in Section 3.1–Section 3.3.

### 3.1. System Setup

**Figure 2:**
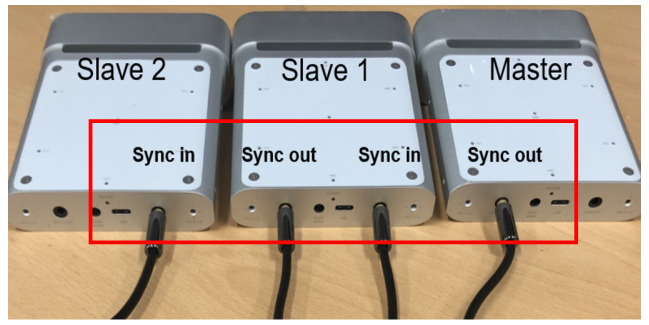
Example of daisy-chain configuration for hardware synchronization.

In order to process the proposed method, the following steps are performed. First, because processing is based on multiple cameras, the RGB-D cameras should be synchronized. The 3.5 mm synchronization in/out plugs are used to synchronize other Azure Kinect DK cameras. In this study, we use a daisy-chain configuration for hardware synchronization. An example of daisy-chain configuration for hardware synchronization is shown in Figure 2.

**Figure 3:**
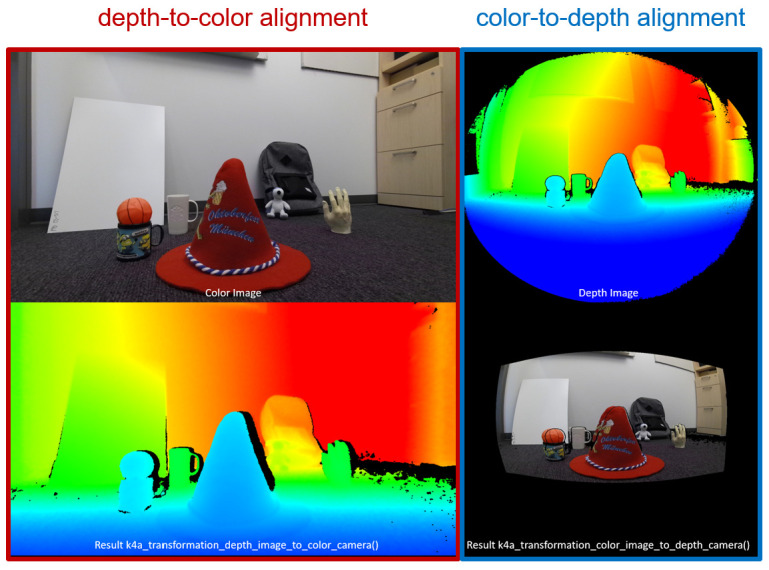
Example of color-depth alignment.

In Figure 2, the daisy-chain configuration for hardware synchronization is a wiring scheme in which multiple Azure Kinect DK cameras are wired together in sequence or in a ring. In this configuration, two factors affect the synchronized cameras. One is the exposure considerations. It is recommended to use a manual exposure setting because under the automatic exposure setting, each color camera can dynamically change the actual exposure. Because the exposure affects the timing, such changes quickly push the cameras out of the synchronization. Therefore, the same exposure setting should be used. Another is avoiding interference between multiple depth cameras. When multiple depth cameras are imaging overlapping fields of view, each camera must image its own associated laser. To prevent the lasers from interfering with one another, the image acquisitions by the cameras should be offset from one another by 160 μs or more. Second, the color and depth images should be aligned. There are color and depth cameras in Azure Kinect DK. As these two cameras are in different positions, the viewpoint of the image acquired by each camera is different. In order to solve this problem, the color-to-depth or depth-to-color alignment should be performed using the intrinsic parameter of each cameras and the extrinsic parameter between the color and depth cameras. The alignment aims to find a one-to-one correspondence between pixels of color and depth images. In order to implement this, three transforms are required: reconstruction, transformation of coordinates and re-projection. The reconstruction transforms 2d pixels into 3d points about the depth camera using intrinsic and distortion parameters of the camera. The transformation of coordinates transforms coordinates of depth camera into coordinates of color camera using extrinsic parameter between depth and color cameras. The re-projection projects 3D points onto 2D pixels about the color camera using intrinsic and distortion parameters of the camera. After all of these transforms, we can find a one-to-one correspondence between pixels of color and depth images. All of these parameters are provided by Azure kinect DK. An example of color–depth alignment is shown in Figure 3.

In Figure 3, the viewpoint of the original color and depth images does not match per pixel. Through the alignment, this problem is solved. However, the depth-to-color alignment causes depth information loss. In this study, we use the color-to-depth alignment to solve this problem. In addition, we apply a bilateral filter to remove the noise from the depth image.

### 3.2. Global Registration Using Human Body Tracking

**Figure 4:**
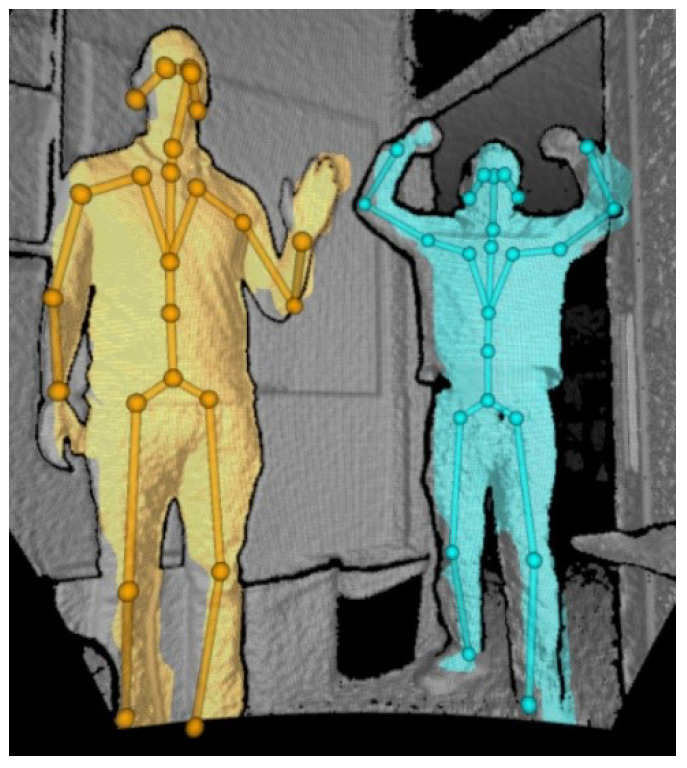
Example of human body tracking. (source: Microsoft).

**Figure 5:**
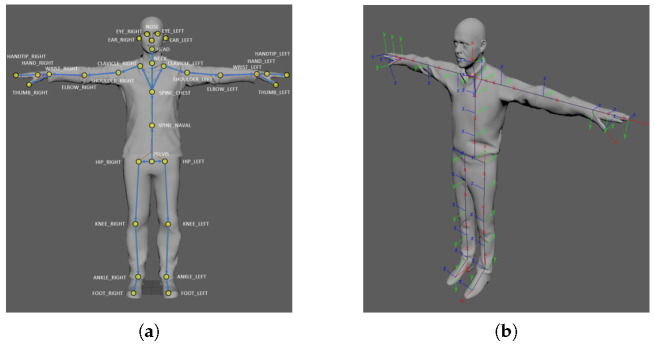
Configuration of the joints. (**a**) Name of each joints, (**b**) Axis orientation of each joint (source: Microsoft).

After the system setup, we process the global registration using deep-learning-model-based human body tracking provided by Azure Kinect SDK. An example of human body tracking is shown in Figure 4. The human body tracking system tracks multiple human bodies simultaneously in the depth image. Each body includes the ID information for temporal correlation between frames and the skeleton. The skeleton contains 32 joints with a joint hierarchy that flows from the center of the body to the extremities. The configuration of the joints and are shown in Figure 5.

**Figure 6:**
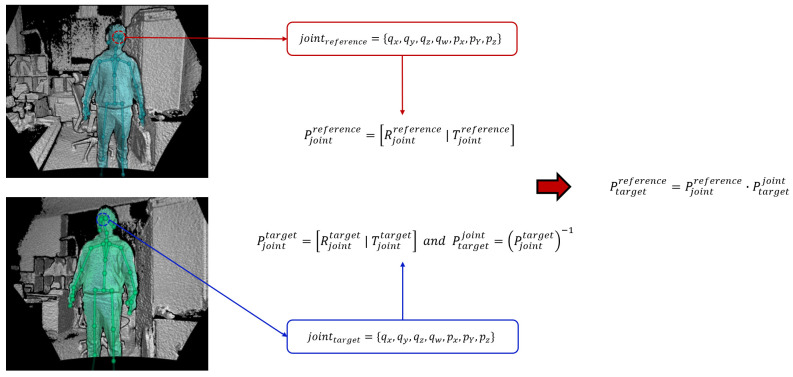
Illustration of proposed global registration.

**Figure 7:**
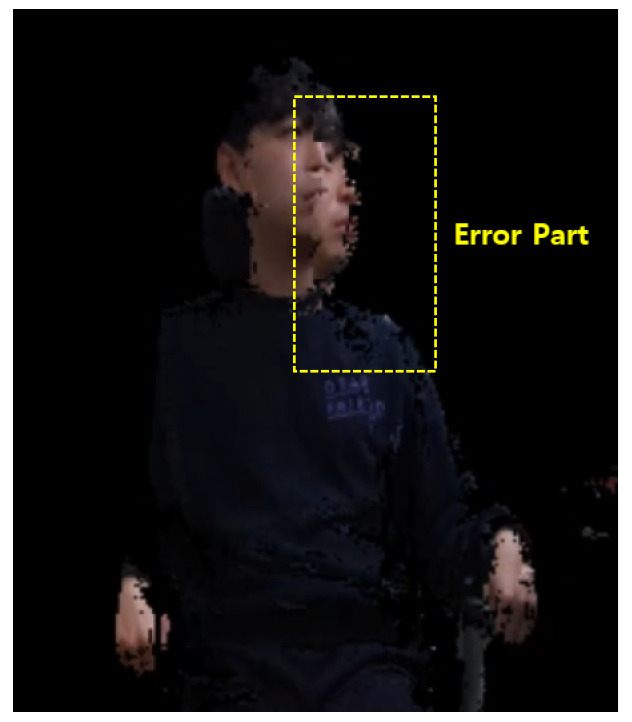
Example of the proposed global registration.

Each joint includes the position, $\left(p_{x},p_{y},p_{z}\right)$, and the axis orientation, $\left(q_{x},q_{y},q_{z},q_{w}\right)$. The joint position and axis orientation are estimated relative to the depth camera. The axis orientation is expressed as a normalized quaternion. In this study, we collect 100 axis orientations and joint positions of the head and chest. This is because the confidence of the head and chest is more precise than others. After collecting the data, we average each item to calculate the global registration. Figure 6 and Figure 7 show an explanation and example of the proposed global registration. In Figure 6, $P_{joint}^{reference},R_{joint}^{reference},T_{joint}^{reference}$ are rigid transform, rotation and translation matrix from the joint to reference, respectively, while $P_{joint}^{target},R_{joint}^{target},T_{joint}^{target}$ represent one from the joint to target. In Figure 7, we can observe that the global registration is obtained with some error. This error should be reduced for a more precise result. In the next section, we propose an iterative method of registration refinement of the global information using feature matching to reduce this error.

### 3.3. Registration Refinement Using Feature Matching

**Figure 8:**
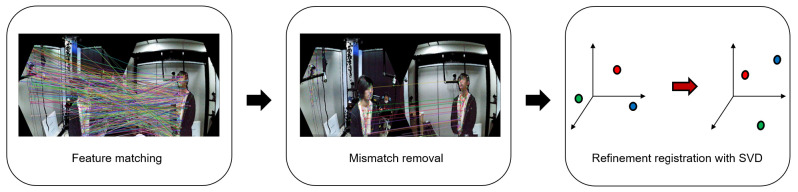
Flowchart of the registration refinement procedure.

After the global registration, we perform registration refinement using feature matching. The flowchart of the registration refinement procedure is shown in Figure 8.

**Figure 9:**
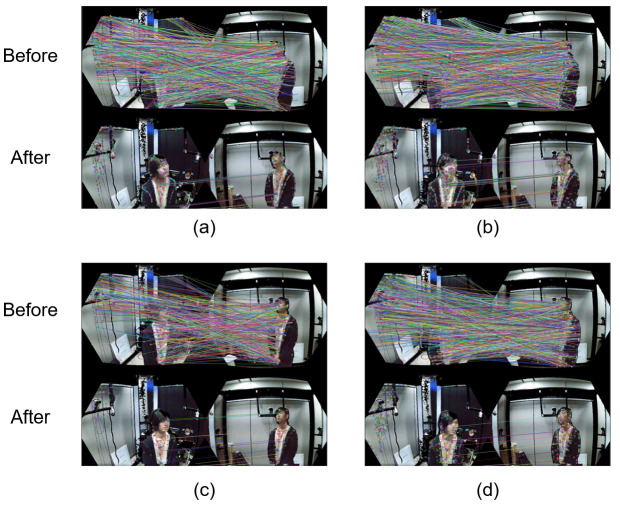
Comparison of the results before and after using the matching pair collection module, (**a**) BRISK, (**b**) ORB, (**c**) SIFT, (**d**) SURF.

The registration refinement system consists of three modules: feature matching, mismatch removal, and refinement registration with singular value decomposition (SVD). In the feature matching module, feature points are matched between each camera. There are many features like SIFT [19], SURF [24], ORB [28], BRISK [25], and so on. In this module, we use BRISK [25], ORB [28], SIFT [19], and SURF [24] features for feature matching between each camera. However, there are mismatches in the set of the matched features. To obtain a more accurate refinement, the mismatches should be removed. The mismatch removal module removes the mismatches generated during feature matching. In order to remove the mismatches, we use the registration to check if features are mismatched. A comparison of the results before and after using the matching pair collection module is shown in Figure 9.

In Figure 9, we process BRISK, ORB, SIFT, and SURF features. As can be seen, there are many matched pairs including good matches and mismatches. The criterion for distinguishing good matches from mismatches is how close the matching points are. Equation (1) is used for removing the mismatches.

(1) $$∥X_{reference}-\left(R_{target}^{reference}\cdot X_{target}+T_{target}^{reference}\right)∥\leq Threshold$$

$X_{reference}$ and $X_{target}$ are 3D pointcloud of the reference and target camera, respectively. $R_{target}^{reference}$ and $T_{target}^{reference}$ are rotation and translation matrix from the target to reference. Threshold indicates a distance threshold for checking mismatches. Using the global registration, matched feature pairs can be transformed into the same coordinate system. After this transformation, we can calculate the L2 distance between each matched pair. Thereafter, if the distance is greater than the threshold, the matched pair is considered to be a mismatch and is eliminated. In this step, the matched pairs consist of good matches. After this step, the refinement registration with SVD module calculates the global registration using the good matches. In this module, we use Umeyama’s method [39] for calculating the global registration between a reference and a target. In [39], the authors infer that it is possible to resolve the least-squares problem between the two point sets by using SVD of a covariance matrix of the data. In this paper, we process this iterative step to obtain more precise global registration results. Equations (2)–(7) represent Umeyama’s method [39]. In Equations (2)–(7), $C_{reference}$ and $C_{target}$ are the centroids of the point sets of each camera, *H* is the covariance matrix between the reference and the target.

(2) $$C_{reference}=\frac{1}{N}\sum_{i=1}^{N}X_{reference}^{i}$$

(3) $$C_{target}=\frac{1}{N}\sum_{i=1}^{N}X_{target}^{i}$$

(4) $$H=\left(X_{target}-C_{target}\right){\left(X_{reference}-C_{reference}\right)}^{T}$$

(5) [U,S,V]=SVD(H)

(6) $$R_{target}^{reference}=V\cdot U^{T}$$

(7) $$T_{target}^{reference}=C_{reference}-R_{target}^{reference}\cdot C_{target}$$

## 4. Experimental Results

**Figure 10:**
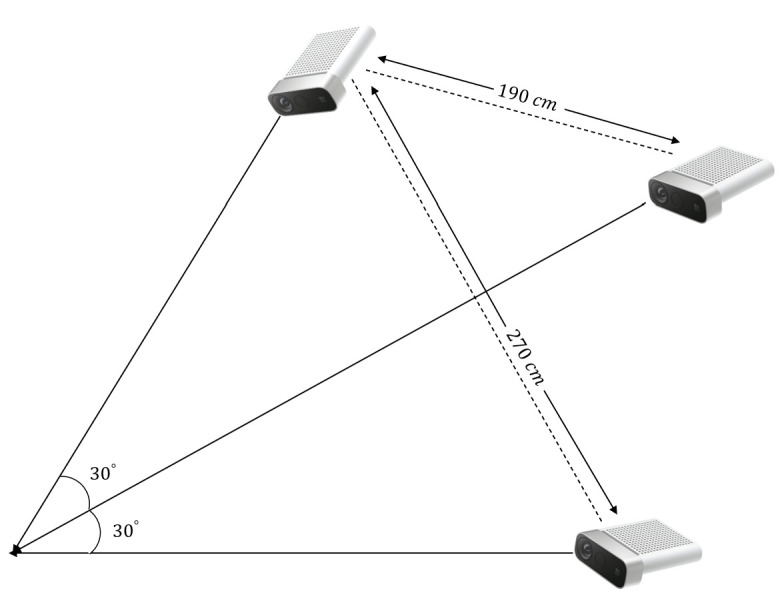
Structure of the experimental environment.

**Figure 11:**
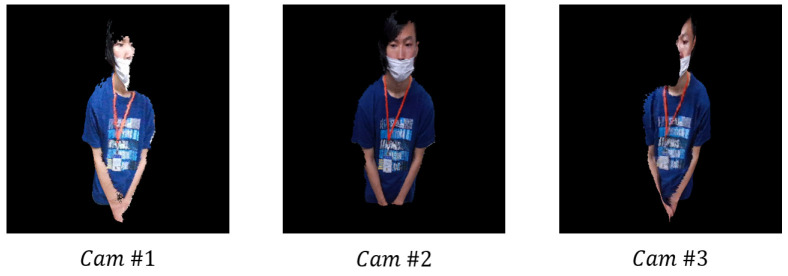
Views of the three cameras used in the experiment.

In this study, we evaluate the proposed method in the experimental environment. We analyze two cases. In the first case, the angle is 30${}^{\circ }$, and the distance is 190 cm between two cameras. In the second case, the angle is 60${}^{\circ }$, and the distance is 270 cm between two cameras. The experimental environment and the views of the three cameras used in the experiment are shown in Figure 10 and Figure 11, respectively.

**Figure 12:**
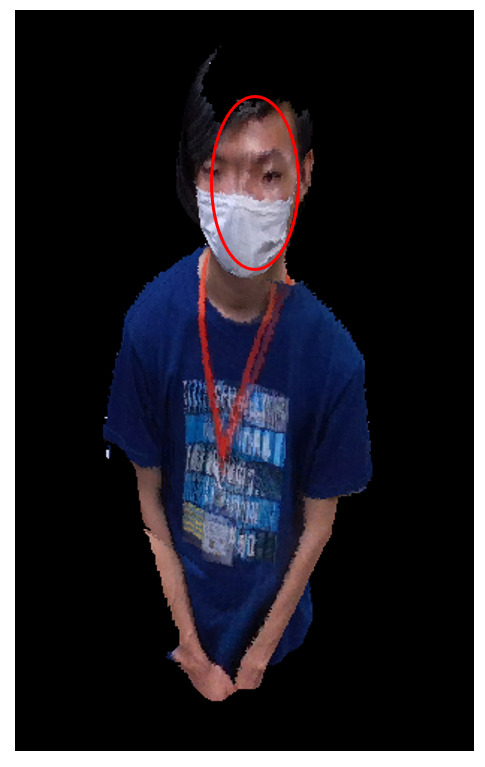
Result of the global registration using human body tracking.

In this environment, a fine global registration is difficult to realize. In this section, we apply the proposed method of the global registration using the human body tracking system and the registration refinement procedure using feature matching to this experimental environment and evaluate the quality though 3D data fusion. There are various methods of 3D data fusion. In this study, we used the truncated signed distance function (TSDF) volume to integrate multiple 3D data. The TSDF volume is the type of volumetric representation of a scene for integrating depth maps. It has the advantages of time and space efficiencies. Several widely used approaches such as KinectFusion [40], fusion4D [41] and Motion2fusion [42] are based on the TSDF volume. The result of the global registration of human body tracking is shown in Figure 12.

As can be seen in Figure 12, the global registration using human body tracking has some error. This error can be reduced with the registration refinement procedure proposed herein. In this study, the proposed method is implemented in several stages. The strategy of the registration refinement is shown in Table 1.

The strategy is composed of six stages. We selected the threshold in each stage based on our experience and figured out that the proposed global registration showed an error of approximately 5 cm. Therefore, we performed the experiments with the threshold decreasing from 5 to 1 cm as shown in Table 1, based on the observation. Stages 1 and 2 are designed with the aim of finding the global rotation and translation, but they contain some mismatches. Stages 3–6 are designed to obtain more details than in the previous stages. By tightening the mismatch removal condition, a more precise inlier can be obtained. For each stage, we implemented feature matching using ORB [28], BRISK [25], SURF [24], and SIFT [19] provided by the OpenCV library. Afterwards, we tested the proposed method with different distance thresholds for each matched feature by counting the inliers among the pairs of the matched features. The test environment was an angle of $30^{\circ }$ and a distance of 190 cm between two cameras for 20 nonconsecutive frames. The result of the proposed registration refinement procedure is shown in Table 2.

In Table 2, the total number of the pairs, number of the adopted pairs used in the proposed method, and ratio of the inliers after utilizing the registration refinement strategy described in Table 1 are shown.

(8) $$The\;ratio\;of\;the\;inliers=1.0-\frac{adopted\;pair\;of\;the\;feature\;matching}{total\;pair\;of\;the\;matched\;features}$$

**Figure 13:**
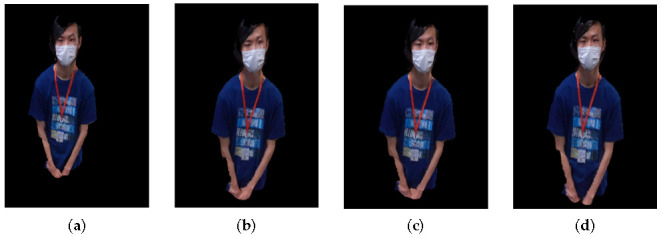
Result of the TSDF fusion using the registration refinement. (**a**) ORB, (**b**) BRISK, (**c**) SIFT, (**d**) SURF.

In Equation (8), the ratio of the inliers is calculated by directly counting the inliers and the mismatches. The numbers of all pairs and adopted pairs used in the proposed method with the SIFT [19] and SURF [24] are lower than in other cases. In addition, the ratio of the inliers for the SIFT [19] is the highest for all distance thresholds. This shows that the SIFT [19] expresses the features within the image well even in difficult situations with considerable rotations and translations. The other features also show inlier ratios of greater than 97% with a distance threshold of 1 mm. This indicates that the proposed method works well for all tested features. The result of the TSDF fusion using the registration refinement of these features is shown in Figure 13.

The result of the TSDF fusion is constructed using the RGB-D information of the three cameras, as shown in Figure 10 and Figure 11. In Figure 13, it is shown that all of the result of the TSDF fusion is clear, as compared to the global registration using human body tracking shown in Figure 12. By using the proposed registration refinement procedure, a more precise global registration of RGB-D cameras can be obtained.

**Figure 14:**
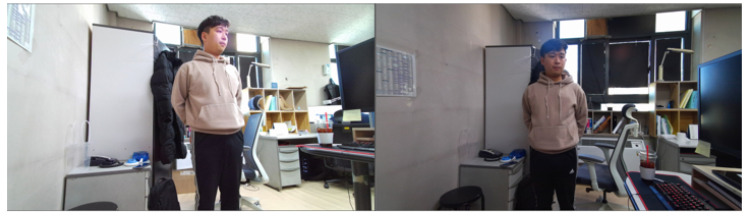
Experimental environment for global registration.

**Figure 15:**
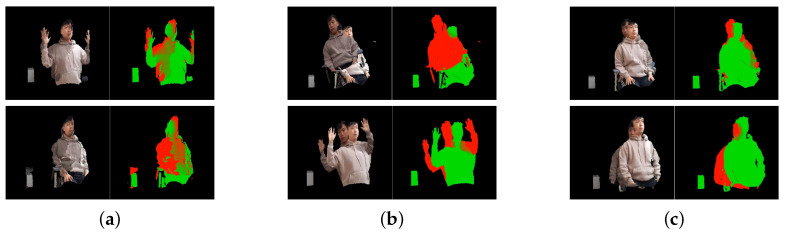
Comparison of the proposed global registration and other methods, (**a**) proposed global registration, (**b**) FPFH and RANSAC, (**c**) fast global registration.

We compare the proposed global registration to other methods. Other methods are fast global registration [9] and global registration based on fast point feature histograms (FPFH) and RANSAC [43]. The experimental environment for global registration is shown in Figure 14. The angle is set to 30${}^{\circ }$, and the distance is set to 80 cm between the two cameras. The test data consist of scenes, in which an individual poses in various poses. The number of the poses is approximately twenty. The comparison of the proposed global registration to other methods is shown in Figure 15. In Figure 15, the left part of each element represents data fused by using the global registration. Meanwhile, the right part of each element shows data visualized in green by a reference camera, and in red by a target camera, respectively. Fast global registration [9] and global registration based on FPFH and RANSAC [43] show the results, which contain a number of error. The proposed global registration has some error, but it shows better performance than other methods.

## 5. Conclusions

In this paper, we proposed a method to fine the global registration between multiple RGB-D cameras. In the first module of the proposed method, a human body tracking system based on deep learning provided by Azure Kinect SDK is used in order to find the global registration between two cameras. This global registration has some error. In the second module of the proposed method, a registration refinement procedure using feature matching is performed. In order to obtain a more precise result, the number of mismatches in the total number of pairs of the matched features should be estimated. In this step, we used the global registration to eliminate mismatches. From the experimental result, we confirmed that mismatches can be eliminated through the proposed method even in difficult situations. By using the proposed method, the global registration between the RGB-D cameras can be obtained effectively. However, this method has the limitation that the initial global registration must be obtained somewhat well. Thus, if we can find an initial global registration that is robust to rotation and translation between two cameras, we can more easily find a more precise global registration.

## Figures and Tables

**Table 1:** Strategy of the registration refinement.

Order of the Stage	Distance Threshold (mm)	Number of the Pairs of the Matched Features
Stage 1	50	200
Stage 2	50	200
Stage 3	20	200
Stage 4	20	100
Stage 5	10	100
Stage 6	10	50

**Table 2:** Result of the proposed registration refinement procedure.

	Distance Threshold (mm)	Total Pair of the Matched Features	Adopted Pair of the Feature Matching with the Proposed Method	Ratio of the Inliers
SIFT [19]	50	1785	387	0.8733
	20		346	0.9768
	10		341	0.9912
SURF [24]	50	1460	313	0.8594
	20		285	0.9438
	10		271	0.9926
BRISK [25]	50	2117	456	0.8333
	20		405	0.9382
	10		386	0.9844
ORB [28]	50	4213	679	0.8600
	20		621	0.9404
	10		597	0.9782
